# Continuity of Care for Patients with Obesity-Associated Chronic Conditions: Protocol for a Multisite Retrospective Cohort Study

**DOI:** 10.2196/20788

**Published:** 2020-09-09

**Authors:** Yilu Lin, James E Bailey, Satya Surbhi, Sohul A Shuvo, Christopher D Jackson, Ming Chen, Eboni G Price-Haywood, Joshua Mann, Daniel Fort, Jeffrey Burton, Ramona Sandlin, Alexandra Castillo, Hao Mei, Patti Smith, Cardella Leak, Phi Le, Alisha M Monnette, Lizheng Shi

**Affiliations:** 1 Department of Health Management and Policy School of Public Health and Tropical Medicine Tulane University New Orleans, LA United States; 2 Center for Health System Improvement, College of Medicine The University of Tennessee Health Science Center Memphis, TN United States; 3 Ochsner Center for Outcomes and Health Services Research New Orleans, LA United States; 4 John D Bower School of Population Health University of Mississippi Medical Center Jackson, MS United States; 5 Center for Informatics and Analytics University of Mississippi Medical Center Jackson, MS United States

**Keywords:** continuity of care, obesity-associated chronic conditions, disparity, obesity, diabetes

## Abstract

**Background:**

Obesity affects nearly half of adults in the United States and is contributing substantially to a pandemic of obesity-associated chronic conditions such as type 2 diabetes, hypertension, and arthritis. The obesity-associated chronic condition pandemic is particularly severe in low-income, medically underserved, predominantly African-American areas in the southern United States. Little is known regarding the impact of geographic, income, and racial disparities in continuity of care on major health outcomes for patients with obesity-associated chronic conditions.

**Objective:**

The aim of this study is to assess, among patients with obesity-associated chronic conditions, and within this group, patients with type 2 diabetes, (1) whether continuity of care is associated with lower overall and potentially preventable emergency department and hospital utilization, (2) the effect of geographic, income, and racial disparities on continuity of care and on health care utilization, (3) whether continuity of care particularly protects individuals at risk for disparities from adverse health outcomes, and (4) whether characteristics of health systems are associated with higher continuity of care and better outcomes.

**Methods:**

Using 2015-2018 data from 4 practice-based research networks participating in the Southern Obesity and Diabetes Coalition, we will conduct a retrospective cohort analysis and distributed meta-analysis. Patients with obesity-associated chronic conditions and with type 2 diabetes will be assessed within each health system, following a standardized study protocol. The primary study outcomes are overall and preventable emergency department visits and hospitalizations. Continuity of care will be calculated at the facility level using a modified version of the Bice-Boxerman continuity of care index. Race will be assessed using electronic medical record data. Residence in a low-income area or a health professional shortage area respectively will be assessed by linking patient residence ZIP codes to the Centers for Medicare & Medicaid Services database.

**Results:**

In 4 regional health systems across Tennessee, Mississippi, Louisiana, and Arkansas, a total of 53 adult hospitals were included in the study. A total of 147,889 patients with obesity-associated chronic conditions who met study criteria were identified in these health systems, of which 45,453 patients met the type 2 diabetes criteria for inclusion. Results are expected by the end of 2020.

**Conclusions:**

This study should reveal whether health system efforts to increase continuity of care for patients with obesity and diabetes have potential to improve outcomes and reduce costs. Analyzing disparities in continuity of care and their effect on major health outcomes can help demonstrate how to improve care and use of health care resources for vulnerable patients with obesity-associated chronic conditions, and within this group, patients with type 2 diabetes. Better understanding of the association between continuity and health care utilization for these vulnerable populations will contribute to the development of higher-value health systems in the southern United States.

**International Registered Report Identifier (IRRID):**

DERR1-10.2196/20788

## Introduction

### Background

Obesity affects nearly half (40%) of adults in the United States [1,2] and contributes substantially to a pandemic of obesity-associated chronic conditions. Obesity commonly leads to multiple chronic conditions such as type 2 diabetes, hypertension, cardiovascular disease, cerebrovascular disease, and arthritis [3]. Our recent research shows that 34% of patients with obesity-associated chronic conditions in the midsouthern United States have diabetes. Projections indicated that 81 million adults will have multimorbidity by 2020, and obesity serves as an underrecognized root cause for this national pandemic of multimorbidity [4-7]. The pandemic of obesity-associated chronic conditions is particularly severe in low-income, medically underserved, and predominantly African-American areas in the southern United States.

Furthermore, the burdens of uncontrolled obesity-associated chronic conditions are rising nationwide, particularly in areas designated by the Human Resources and Services Administration as primary care health professional shortage areas of the southern United States [8-10]. The economic burden of adult obesity on the United States exceeds $215 billion [11]; the most common obesity-associated chronic conditions are known to increase health care resource utilization and costs [12-15]. Access to regular primary and specialty care is associated with improved chronic care management and reduces potentially preventable health care utilization attributed to obesity-associated chronic conditions [16,17]. Prior evidence from observational studies suggests that continuity of care is associated with higher medication adherence and lower health care resource utilization among older adults and patients with multiple chronic conditions [18-24]. But little is known regarding the impact of continuity of care for patients with type 2 diabetes or other obesity-associated chronic conditions in medically underserved areas of the southern United States.

There is a critical need to quantify the impact of geographic, income, and racial disparities on continuity of care and health care utilization and identify characteristics of health systems with higher continuity and lower health care utilization for patients with obesity-associated chronic conditions and type 2 diabetes. Effective population health improvement requires examining comprehensive data across health systems to look at variations in care. Such pragmatic research has the potential to translate best health systems practices across different communities in the southern United States.

### Objectives

This protocol aims to assess (1) whether outpatient continuity of care is associated with lower overall and potentially preventable emergency department and hospital utilization for patients with obesity-associated chronic conditions and with type 2 diabetes, respectively; (2) the effect of geographic, income, ethnic, and racial disparities on continuity of care and overall and potentially preventable emergency department and hospital utilization among patients with obesity-associated chronic conditions and with type 2 diabetes; (3) whether continuity of care particularly protects individuals with obesity-associated chronic conditions and with type 2 diabetes at risk for geographic, income, ethnic, and racial disparities from overall and potentially preventable hospital and emergency department utilization; and (4) the characteristics of health systems with higher continuity and lower health care utilization for vulnerable patients with obesity-associated chronic conditions and in the type 2 diabetes.

We hypothesize that continuity of care will protect vulnerable individuals with obesity-associated chronic conditions, and within that group, individuals with type 2 diabetes, from overall and potentially preventable emergency department and hospital utilization. Additionally, continuity of care will be associated with lower emergency department and hospital use for (1) African-American individuals compared with that of white individuals or other individuals, (2) patients residing in health professional shortage areas compared with that of patients not residing in health professional shortage areas, and (3) patients residing in low-income areas compared with that of patients not residing in low-income areas. We also hypothesize that health systems with (1) a higher proportion of primary care providers to total providers, (2) a higher proportion of ambulatory to total encounters, and (3) a greater geographic distribution of ambulatory care sites will experience lower levels of overall and potentially preventable emergency department and hospital use among patients receiving ambulatory care within the system.

## Methods

### Study Design

We will conduct a retrospective cohort analysis and meta-analysis of all adult patients with obesity-associated chronic conditions—defined as patients who are obese (BMI≥30 kg/m^2^) and who have ≥1 additional diagnosed obesity-associated chronic condition (a list of the included conditions is available in Multimedia Appendix 1) [25,26]—who are seen in health systems and practice-based research networks participating in the Southern Obesity and Diabetes Coalition.

**Figure 1:**
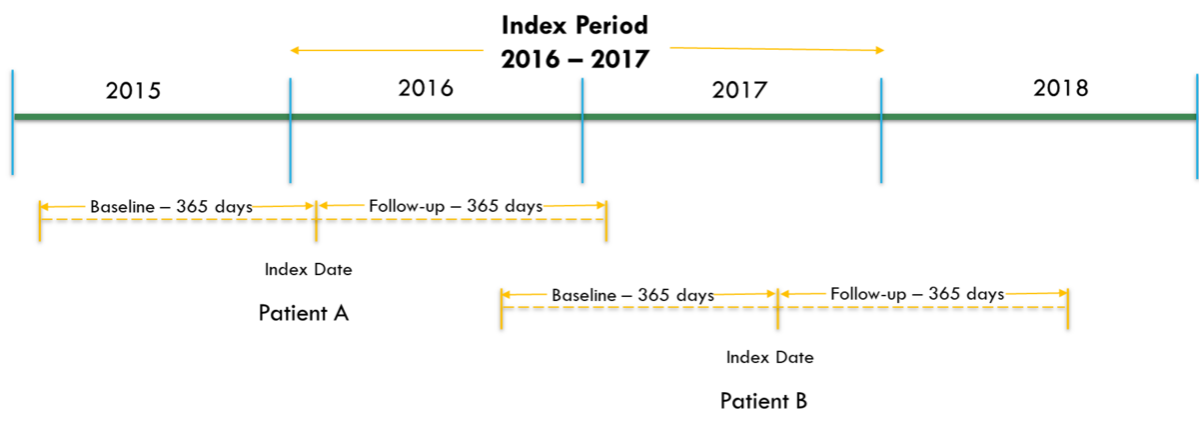
Study design.

This study will be conducted separately at the following sites: (1) University of Tennessee Health Science Center, (2) University of Mississippi Medical Center, (3) Tulane Medical Center, and (4) Ochsner Health System. The data from all sites will be aggregated to conduct meta-analysis. Electronic medical records from between 2015 and 2018 including encounters from all adult emergency department, hospitals, and clinics will be utilized. The index visit will be defined as the first visit of any type occurring between January 1, 2016 and December 31, 2017. The baseline year will be defined as the first full year prior to the qualifying index visit date (as defined above) for which the full inclusion criteria are met. The baseline year will be used to identify adult patients with obesity-associated chronic conditions and type 2 diabetes seen 2 or more times for ambulatory care. Outcomes will be assessed in a 1-year period following the index visit. The study design is shown in Figure 1.

### Participant Inclusion and Exclusion Criteria

The study will include patients with obesity-associated chronic conditions and with type 2 diabetes who meet the following criteria: patients aged >18 years at their index visit, who are obese (defined as BMI>30 kg/m^2^) at their index visit (if BMI>100 kg/m^2^, they will be excluded; if there are multiple valid BMI values associated with the index visit, the mean BMI values will be used), with ≥1 diagnosed obesity-associated chronic conditions at any time in any location in their baseline year (see Multimedia Appendix 1 for definition of obesity-associated chronic conditions and qualifying ICD-9-CM and ICD-10-CM diagnosis codes) [25,26], with > 2 ambulatory visits in the baseline year, and with >1 encounter of any type in the follow-up year. Of those, patients with diagnosed type 2 diabetes at any time in any location in their baseline year will be identified.

### Outcome Variables

Outcome variables will include overall emergency department visits and overall hospitalizations. These are 2 of the 4 primary outcomes for outpatient continuity of care association with health care use; the effect of geographic, income, ethnic, and racial disparities; and continuity of care protective effects; and are secondary outcomes for characteristics of health care systems with high continuity of care and low utilization. Hospitalizations will include observations visits, and emergency department visits resulting in observation or inpatient visits on the same or following day will be counted as hospitalizations.

Preventable emergency department visits and preventable hospitalizations will be defined by The Agency for Healthcare Research and Quality Prevention Quality Indicators chronic composite measures and will be identified using the primary diagnosis for diabetes short-term complications, diabetes long-term complications, chronic obstructive pulmonary disease or asthma in older adults, hypertension, angina without procedure, uncontrolled diabetes, asthma in younger adults, and lower extremity amputation among patients with diabetes [27]. Hospitalizations will include observations visits. Emergency department visits resulting in observation or inpatient visits on the same or following day will be counted as hospitalizations.

### Independent Variables

*Continuity of care* will be assessed for each patient using a modified version of the Bice-Boxerman index to assess continuity of care at the clinic or facility level [18,19,28]. The continuity of care index is a measure of concentration of the patient-clinic visit patterns ranging from 0 (each visit involved a different practice) to 1 (all visits were made to a single practice). It reflects the relative share of all of a patient’s visits during the year that were made to distinct practices. Thus, the higher the index score, the better the modified continuity of care. In this study, a modified Bice-Boxerman continuity of care index score will be used to assess the concentration of the patient-clinic visits patterns during the 12-month baseline period. For this measure, a patient-clinic visit is defined as an encounter with a unique combination of admit date, facility ID (or clinic name), and an ambulatory encounter type. The measure will include both primary and specialty care visits and is calculated as:

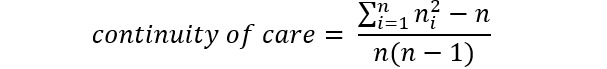



[18,19,28]

*Low-income status* will be assessed at the individual level using patient residence in a low-income area. Patient residence ZIP codes will be linked to the Centers for Medicare & Medicaid Services database of health professional shortage area and low-income ZIP codes to identify patients residing in low-income areas [29].

*Residence* in health professional shortage area will also be assessed at the individual level using patient residence ZIP code linkage to the Centers for Medicare & Medicaid Services database of health professional shortage area and low-income ZIP codes as described above [29].

A *health system* will be defined to include all ambulatory sites and inpatient facilities included in each respective practice-based research networks. For each health system so defined, the following measures will be calculated using available practice-based research networks and health system data:

Percentage of total providers with primary care specialty (number unique primary care providers / total unique providers), using “ProviderID” and “Provider_Specialty_Primary” labels from practice-based research networks data or, if necessary, using health system provider records.

Percentage of total encounters in ambulatory setting (number of ambulatory encounters / number of ambulatory + hospital encounters) in the practice-based research networks.

Ambulatory site geographic density (number of ambulatory sites per square mile for all unique ZIP codes with one or more health system ambulatory sites or inpatient facilities). We will use the “Facility_Location” label to assess ZIP codes for clinic sites, or if necessary, health system records.

Other covariates such as sociodemographic factors will be assessed at the index visit or the first visit in the baseline year for which data is available and will include age (continuous), gender (male, female), and race (Black or African-American, White, other). Clinical factors will include Charlson comorbidity index [30], and diagnosis of anxiety or depression assessed in the baseline period.

### Statistical Analysis

The main analysis will be conducted among patients with obesity-associated chronic conditions. Multivariable negative binomial models will be used to model overall and preventable hospitalizations and emergency department visits. For continuity of care as an outcome, a fractional regression model will be used. Interaction terms between (1) continuity of care and patient residence health professional shortage area designation, (2) continuity of care and patient residence low income status, and (3) continuity of care and patient race will be included to examine whether the association between continuity and health care utilization varies by these factors. The same will be assessed in patients with type 2 diabetes.

Finally, we will conduct a distributed meta-analysis using direct aggregate data from each site following the method employed by Toh [31,32] and Zhou and colleagues [33] to integrate study findings from the 4 different study sites. This approach employs inverse variance-weighted meta-analysis using multivariable confounding adjustment across distributed data networks without sharing of patient-level data [32].

### Ethics and Dissemination

This study protocol has been approved by the Institutional Review Board of the 4 sponsoring institutions (University of Tennessee Health Science Center protocol 18-06394-XP, Tulane Medical Center protocol 2018-2306, University of Mississippi Medical Center protocol 2019-0005, Ochsner Health System protocol 2019.035). The findings of this study will be submitted as manuscripts to peer-reviewed journals to aid dissemination to policy makers and other researchers in the field. It will also be presented and discussed at Annual Delta Clinical and Translational Science Health Disparities Conference.

## Results

Each site is currently analyzing data, reporting, and submitting the aggregate results; 4 regional health systems across the southern United States have completed collection of data sets and data management at each site. Table 1 shows the characteristics with each health system by demonstrating their geographic characteristics (city, state, and geographic coverage) and hospital characteristics (number of adult hospitals, number of outpatient practices, and number of unique adult patients served). Out of 2,155,860 adult patients served by 53 hospitals and over 400 outpatient practices in the 4 health systems representing Tennessee, Mississippi, Louisiana, and Arkansas, we found that 147,889 patients had obesity-associated chronic conditions and 45,453 patients had type 2 diabetes. Combining results from all sites, data management, and analysis of combined data will be completed by July 2020. Dissemination is expected by the end of 2020.

**Table 1:** Southern Obesity and Diabetes Coalition practice-based research network locations and characteristics.

Sponsoring institution	City and state	Geographic coverage	Adult hospitals, n	Outpatient practices, n	Unique adult patients served, n
Ochsner Health System	Jefferson, Louisiana	Southeast Louisiana including the Greater New Orleans area, Slidell, Covington, Raceland, and Baton Rouge	40	>100	1,374,751
Tulane Medical Center	New Orleans, Louisiana	Greater New Orleans area	4	34	25,709
University of Mississippi Medical Center	Jackson, Mississippi	Mississippi	4	255	361,288
University of Tennessee Health Science Center	Memphis, Tennessee	West Tennessee, Arkansas, Mississippi	5	60	394,112

## Discussion

The 4 health systems and their respective practice-based research networks include patients across 4 southern states, including Arkansas, Louisiana, Mississippi, and Tennessee, with among the highest obesity prevalence in the United States at 37.1%, 36.8%, 39.5% and 34.4%, respectively [1]. Previous studies indicate that health system efforts to increase continuity of primary care for patients with obesity-associated chronic conditions have the potential to improve outcomes and reduce costs [34,35]. There is a critical need to quantify the impact of geographic, income, and racial disparities on continuity of care and health care utilization for people living with obesity-associated chronic conditions and to identify potentially modifiable health system characteristics associated with higher continuity of care and lower health care utilization for these highly vulnerable populations. This study will inform ongoing health system improvement across the southern United States and could lead to major funding for pragmatic research to help communities most affected by disparities to invest in health system transformation to reduce disparities and improve health.

Our research employs an innovative method—inverse variance-weighted meta-analysis across distributed data networks—as employed by the FDA minisentinel program and others [31-33]. The most desirable feature of this approach is that the research process could be easily managed by individual sites and there is no need to share the data with other sites. Each site generates their own outcome results and these outcomes will be synthesized into one treatment effect. As long as each site follows the same research principles and protocol guidelines, this approach will enable our newly established Southern Obesity and Diabetes Coalition and its Cooperative Meta-Analysis Group to immediately support studies across a variety of chronic conditions. Study investigators collaborated to develop standardized protocol to harmonize data collection, management, and analyses across different health systems with differing underlying populations and data sources. This study will assess a very large study cohort with substantial geographic diversity and will provide important information regarding factors impacting health care for patients with obesity-associated chronic conditions. The study will address key knowledge gaps regarding the impact of geographic, income, and racial disparities on continuity of care and major health outcomes for individuals with obesity-associated chronic conditions and with type 2 diabetes.

The study is subject to some limitations. Although we standardized data attributes to the best of our ability, there were still a few variables that were not consistently reported across the sites. As a result, we were unable to assess insurance status, provider-level continuity of care, primary-care continuity of care, and specialty-care continuity of care due to lack of consistent information. The study is also limited by inconsistencies in data collection practices among participating providers. For example, the income and race or ethnicity information were not consistently collected for subset populations across health systems.

This study employs an innovative collaborative research approach and has potential to yield important actionable results that can be employed to improve health care delivery for the vulnerable populations with obesity-associated chronic conditions and with type 2 diabetes. The Southern Obesity and Diabetes Coalition’s use of a cooperative meta-analysis method provides a way for researchers to gain the economies of scale needed to answer important pragmatic health services research questions. Furthermore, the worldwide pandemic of obesity and obesity-associated chronic conditions makes this research particularly relevant.

## Appendix

Multimedia Appendix 1 Obesity-associated chronic conditions qualifying diagnosis codes (ICD-9 and ICD-10).

## Abbreviations

FDA: Food and Drug Administration
ICD: International Classification of Diseases
