# Supplementary material for: Continuity of Care for Patients with Obesity-Associated Chronic Conditions: Protocol for a Multisite Retrospective Cohort Study
Source: JMIR Res Protoc. 2020 Sep 9;9(9):e20788. doi: 10.2196/20788 (PMC7511855; doi:10.2196/20788)
Supplement: Multimedia Appendix 1 [file resprot_v9i9e20788_app1.docx]

**Appendix 1: OCC qualifying diagnosis codes (ICD-9 and ICD-10)**

Acute myocardial infarction: '410.01', '410.11', '410.21', '410.31', '410.41', '410.51', '410.61', '410.71', '410.81', '410.91', 'I21.01', 'I21.02', 'I21.09', 'I21.11', 'I21.19', 'I21.21', 'I21.29', 'I21.3', 'I21.4', 'I21.9', 'I21.A1', 'I21.A9', 'I22.0', 'I22.1', 'I22.2', 'I22.8', 'I22.9'

Alzheimer’s disease and related disorders or senile dementia: '331.0', '331.11', '331.19', '331.2', '331.7', '290.0', '290.10', '290.11', '290.12', '290.13','290.20', '290.21', '290.3', '290.40', '290.41', '290.42', '290.43', '294.0', '294.10', '294.11', '294.20', '294.21', '294.8', '797', 'F01.50', 'F01.51', 'F02.80', 'F02.81', 'F03.90', 'F03.91', 'F04', 'G13.8', 'F05', 'F06.1', 'F06.8', 'G30.0', 'G30.1', 'G30.8', 'G30.9', 'G31.1', 'G31.2', 'G31.01', 'G31.09', 'G94', 'R41.81', 'R54'

Asthma: '493.00', '493.01', '493.02', '493.10', '493.11', '493.12', '493.20', '493.21', '493.22', '493.81', '493.82', '493.90', '493.91', '493.92', 'J45.20', 'J45.21', 'J45.22', 'J45.30', 'J45.31', 'J45.32', 'J45.40', 'J45.41', 'J45.42', 'J45.50', 'J45.51', 'J45.52', 'J45.901', 'J45.902', 'J45.909', 'J45.990', 'J45.991', 'J45.998'

Atrial fibrillation: '427.31', 'I48.0', 'I48.1', 'I48.2', 'I48.91'

Benign prostatic hyperplasia: '600.00', '600.01', '600.10', '600.11', '600.20', '600.21', '600.3', '600.90', '600.91', 'N40.0', 'N40.1', 'N40.2', 'N40.3', 'N42.83' (*If any of the qualifying claims also have an ICD-9 diagnosis of 222.2 or and ICD-10 diagnosis of D29.1, then exclude*)

Breast cancer: '174.0', '174.1', '174.2', '174.3', '174.4', '174.5', '174.6', '174.8', '174.9', '175.0', '175.9', '233.0', 'V10.3', 'C50.011', 'C50.012', 'C50.019', 'C50.021', 'C50.022', 'C50.029', 'C50.111', 'C50.112', 'C50.119', 'C50.121', 'C50.122', 'C50.129', 'C50.211', 'C50.212', 'C50.219', 'C50.221', 'C50.222', 'C50.229', 'C50.311', 'C50.312', 'C50.319', 'C50.321', 'C50.322', 'C50.329', 'C50.411', 'C50.412', 'C50.419', 'C50.421', 'C50.422', 'C50.429', 'C50.511', 'C50.512', 'C50.519', 'C50.521', 'C50.522', 'C50.529', 'C50.611', 'C50.612', 'C50.619', 'C50.621', 'C50.622', 'C50.629', 'C50.811', 'C50.812', 'C50.819', 'C50.821', 'C50.822', 'C50.829', 'C50.911', 'C50.912', 'C50.919', 'C50.921', 'C50.922', 'C50.929', 'D05.00', 'D05.01', 'D05.02', 'D05.10', 'D05.11', 'D05.12', 'D05.80', 'D05.81', 'D05.82', 'D05.90', 'D05.91', 'D05.92', 'Z85.3'

Colorectal cancer: '153.0', '153.1', '153.2', '153.3', '153.4', '153.5', '153.6', '153.7', '153.8', '153.9','154.0','154.1', '230.3', '230.4', 'V10.05', 'V10.06', 'C18.0', 'C18.1', 'C18.2', 'C18.3', 'C18.4', 'C18.5', 'C18.6', 'C18.7', 'C18.8', 'C18.9', 'C19', 'C20', 'D01.0', 'D01.1', 'D01.2', 'Z85.038', 'Z85.040', 'Z85.048'

Prostate cancer: '185','233.4', 'V10.46', 'C61', 'D07.5', 'Z85.46'

Endometrial cancer: '182.0', '233.2', 'V10.42', 'C54.1', 'C54.2', 'C54.3', 'C54.8', 'C54.9', 'D07.0', 'Z85.42'

Cataract: '366.01', '366.02', '366.03', '366.04', '366.09', '366.10', '366.12', '366.13', '366.14', '366.15', '366.16', '366.17', '366.18', '366.19', '366.20', '366.21', '366.22', '366.23', '366.30', '366.45', '366.46', '366.50', '366.51', '366.52', '366.53', '366.8', '366.9', '379.26', '379.31', '379.39', '743.30', '743.31', '743.32', '743.33', 'V43.1', 'H25.011', 'H25.012', 'H25.013', 'H25.019', 'H25.031', 'H25.032', 'H25.033', 'H25.039', 'H25.041', 'H25.042', 'H25.043', 'H25.049', 'H25.091', 'H25.092', 'H25.093', 'H25.099', 'H25.10', 'H25.11', 'H25.12', 'H25.13', 'H25.20', 'H25.21', 'H25.22', 'H25.23', 'H25.811', 'H25.812', 'H25.813', 'H25.819', 'H25.89', 'H25.9', 'H26.011', 'H26.012', 'H26.013', 'H26.019', 'H26.031', 'H26.032', 'H26.033', 'H26.039', 'H26.041', 'H26.042', 'H26.043', 'H26.049', 'H26.051', 'H26.052', 'H26.053', 'H26.059', 'H26.061', 'H26.062', 'H26.063', 'H26.069', 'H26.09', 'H26.101', 'H26.102', 'H26.103', 'H26.109', 'H26.111', 'H26.112', 'H26.113', 'H26.119', 'H26.121', 'H26.122', 'H26.123', 'H26.129', 'H26.131', 'H26.132', 'H26.133', 'H26.139', 'H26.20', 'H26.211', 'H26.212', 'H26.213', 'H26.219', 'H26.30', 'H26.31', 'H26.32', 'H26.33', 'H26.40', 'H26.411', 'H26.412', 'H26.413', 'H26.419', 'H26.491', 'H26.492', 'H26.493', 'H26.499', 'H26.8', 'H26.9', 'Q12.0', 'Z96.1'

Chronic kidney disease: '016.00', '016.01', '016.02', '016.03', '016.04', '016.05', '016.06', '095.4', '189.0', '189.9', '223.0', '236.91', '249.40', '249.41', '250.40', '250.41', '250.42', '250.43', '271.4', '274.10', '283.11', '403.01', '403.11', '403.91', '404.02', '404.03', '404.12', '404.13', '404.92', '404.93', '440.1', '442.1', '572.4', '580.0', '580.4', '580.81', '580.89', '580.9', '581.0', '581.1', '581.2', '581.3', '581.81', '581.89', '581.9', '582.0', '582.1', '582.2', '582.4', '582.81', '582.89', '582.9', '583.0', '583.1', '583.2', '583.4', '583.6', '583.7', '583.81', '583.89', '583.9', '584.5', '584.6', '584.7', '584.8', '584.9', '585.1', '585.2', '585.3', '585.4', '585.5', '585.6', '585.9', '586', '587', '588.0', '588.1', '588.81', '588.89', '588.9', '591', '753.12', '753.13', '753.14', '753.15', '753.16', '753.17', '753.19', '753.20', '753.21', '753.22', '753.23', '753.29', '794.4', 'A18.11', 'A52.75', 'B52.0', 'C64.1', 'C64.2', 'C64.9', 'C68.9', 'D30.00', 'D30.01', 'D30.02', 'D41.00', 'D41.01', 'D41.02', 'D41.10', 'D41.11', 'D41.12', 'D41.20', 'D41.21', 'D41.22', 'D59.3', 'E08.21', 'E08.22', 'E08.29', 'E08.65', 'E09.21', 'E09.22', 'E09.29', 'E10.21', 'E10.22', 'E10.29', 'E10.65', 'E11.21', 'E11.22', 'E11.29', 'E11.65', 'E13.21', 'E13.22', 'E13.29', 'E74.8', 'I12.0', 'I12.9', 'I13.0', 'I13.10', 'I13.11', 'I13.2', 'I70.1', 'I72.2', 'K76.7', 'M10.30', 'M10.311',' M10.312', 'M10.319', 'M10.321', 'M10.322', 'M10.329', 'M10.331', 'M10.332', 'M10.339', 'M10.341', 'M10.342', 'M10.349', 'M10.351', 'M10.352', 'M10.359', 'M10.361', 'M10.362', 'M10.369', 'M10.371', 'M10.372', 'M10.379', 'M10.38', 'M10.39', 'M32.14', 'M32.15', 'M35.04', 'N00.0', 'N00.1', 'N00.2', 'N00.3', 'N00.4', 'N00.5', 'N00.6', 'N00.7', 'N00.8', 'N00.9', 'N01.0', 'N01.1', 'N01.2', 'N01.3', 'N01.4', 'N01.5', 'N01.6', 'N01.7', 'N01.8', 'N01.9', 'N02.0', 'N02.1', 'N02.2', 'N02.3', 'N02.4', 'N02.5', 'N02.6', 'N02.7', 'N02.8', 'N02.9', 'N03.0', 'N03.1', 'N03.2', 'N03.3', 'N03.4', 'N03.5', 'N03.6', 'N03.7', 'N03.8', 'N03.9', 'N04.0', 'N04.1', 'N04.2', 'N04.3', 'N04.4', 'N04.5', 'N04.6', 'N04.7', 'N04.8', 'N04.9', 'N05.0', 'N05.1', 'N05.2', 'N05.3', 'N05.4', 'N05.5', 'N05.6', 'N05.7', 'N05.8', 'N05.9', 'N06.0', 'N06.1', 'N06.2', 'N06.3', 'N06.4', 'N06.5', 'N06.6', 'N06.7', 'N06.8', 'N06.9', 'N07.0', 'N07.1', 'N07.2', 'N07.3', 'N07.4', 'N07.5', 'N07.6', 'N07.7', 'N07.8', 'N07.9', 'N08', 'N13.1', 'N13.2', 'N13.30', 'N13.39', 'N14.0', 'N14.1', 'N14.2', 'N14.3', 'N14.4', 'N15.0', 'N15.8', 'N15.9', 'N16', 'N17.0', 'N17.1', 'N17.2', 'N17.8', 'N17.9', 'N18.1', 'N18.2', 'N18.3', 'N18.4', 'N18.5', 'N18.6', 'N18.9', 'N19', 'N25.0', 'N25.1', 'N25.81', 'N25.89', 'N25.9', 'N26.1', 'N26.9', 'Q61.02', 'Q61.11', 'Q61.19', 'Q61.2', 'Q61.3', 'Q61.4', 'Q61.5', 'Q61.8', 'Q62.0', 'Q62.2', 'Q62.10', 'Q62.11', 'Q62.12', 'Q62.31', 'Q62.32', 'Q62.39', 'R94.4'

Depression: '296.20', '296.21', '296.22', '296.23', '296.24', '296.25', '296.26', '296.30', '296.31', '296.32', '296.33', '296.34', '296.35', '296.36', '296.51', '296.52', '296.53', '296.54', '296.55', '296.56', '296.60', '296.61', '296.62', '296.63', '296.64', '296.65', '296.66', '296.89', '298.0', '300.4', '309.1', '311', 'F31.30', 'F31.31', 'F31.32', 'F31.4', 'F31.5', 'F31.60', 'F31.61', 'F31.62', 'F31.63', 'F31.64', 'F31.75', 'F31.76', 'F31.77', 'F31.78', 'F31.81', 'F32.0', 'F32.1', 'F32.2', 'F32.3', 'F32.4', 'F32.5', 'F32.9', 'F33.0', 'F33.1', 'F33.2', 'F33.3', 'F33.40', 'F33.41', 'F33.42', 'F33.8', 'F33.9', 'F34.1', 'F43.21', 'F43.23'

Glaucoma: '362.85', '365.00', '365.01', '365.02', '365.03', '365.04', '365.10', '365.11', '365.12', '365.13', '365.15', '365.20', '365.21', '365.22', '365.23', '365.24', '365.31', '365.32', '365.41', '365.42', '365.43', '365.51', '365.52', '365.59', '365.60', '365.61', '365.62', '365.63', '365.64', '365.65', '365.81', '365.82', ‘365.83', '365.89', '365.9', '377.14', 'H40.001', 'H40.002', 'H40.003', 'H40.009', 'H40.011', 'H40.012', 'H40.013', 'H40.019', 'H40.031', 'H40.032', 'H40.033', 'H40.039', 'H40.041', 'H40.042', 'H40.043', 'H40.049', 'H40.051', 'H40.052', 'H40.053', 'H40.059', 'H40.10X0', 'H40.10X1', 'H40.10X2', 'H40.10X3', 'H40.10X4', 'H40.11X0', 'H40.1110', 'H40.11X1', 'H40.1111', 'H40.11X2', 'H40.1112', 'H40.11X3', 'H40.1113', 'H40.11X4', 'H40.1114', 'H40.1120', 'H40.1121', 'H40.1122', 'H40.1123', 'H40.1124', 'H40.1130', 'H40.1131', 'H40.1132', 'H40.1133', 'H40.1134', 'H40.1190', 'H40.1191', 'H40.1192', 'H40.1193', 'H40.1194', 'H40.1210', 'H40.1211', 'H40.1212', 'H40.1213', 'H40.1214', 'H40.1220', 'H40.1221', 'H40.1222', 'H40.1223', 'H40.1224', 'H40.1230', 'H40.1231', 'H40.1232', 'H40.1233', 'H40.1234', 'H40.1290', 'H40.1291', 'H40.1292', 'H40.1293', 'H40.1294', 'H40.1310', 'H40.1311', 'H40.1312', 'H40.1313', 'H40.1314', 'H40.1320', 'H40.1321', 'H40.1322', 'H40.1323', 'H40.1324', 'H40.1330', 'H40.1331', 'H40.1332', 'H40.1333', 'H40.1334', 'H40.1390', 'H40.1391', 'H40.1392', 'H40.1393', 'H40.1394', 'H40.1410', 'H40.1411', 'H40.1412', 'H40.1413', 'H40.1414', 'H40.1420', 'H40.1421', 'H40.1422', 'H40.1423', 'H40.1424', 'H40.1430', 'H40.1431', 'H40.1432', 'H40.1433', 'H40.1434', 'H40.1490', 'H40.1491', 'H40.1492', 'H40.1493', 'H40.1494', 'H40.151', 'H40.152', 'H40.153', 'H40.159', 'H40.20X0', 'H40.20X1', 'H40.20X2', 'H40.20X3', 'H40.20X4', 'H40.211', 'H40.212', 'H40.213', 'H40.219', 'H40.2210', 'H40.2211', 'H40.2212', 'H40.2213', 'H40.2214', 'H40.2220', 'H40.2221', 'H40.2222', 'H40.2223', 'H40.2224', 'H40.2230', 'H40.2231', 'H40.2232', 'H40.2233', 'H40.2234', 'H40.2290', 'H40.2291', 'H40.2292', 'H40.2293', 'H40.2294', 'H40.231', 'H40.232', 'H40.233', 'H40.239', 'H40.241', 'H40.242', 'H40.243', 'H40.249', 'H40.30X0', 'H40.30X1', 'H40.30X2', 'H40.30X3', 'H40.30X4', 'H40.31X0', 'H40.31X1', 'H40.31X2', 'H40.31X3', 'H40.31X4', 'H40.32X0', 'H40.32X1', 'H40.32X2', 'H40.32X3', 'H40.32.X4', 'H40.33X0', 'H40.33X1', 'H40.33X2', 'H40.33X3', 'H40.33X4', 'H40.40X0', 'H40.40X1', 'H40.40X2', 'H40.40X3', 'H40.40X4', 'H40.41X0', 'H40.41X1', 'H40.41X2', 'H40.41X3', 'H40.41X4', 'H40.42X0', 'H40.42X1', 'H40.42X2', 'H40.42X3', 'H40.42X4', 'H40.43X0', 'H40.43X1', 'H40.43X2', 'H40.43X3', 'H40.43X4', 'H40.50X0', 'H40.50X1', 'H40.50X2', 'H40.50X3', 'H40.50X4', 'H40.51X0', 'H40.51X1', 'H40.51X2', 'H40.51X3', 'H40.51X4', 'H40.52X0', 'H40.52X1', 'H40.52X2', 'H40.52X3', 'H40.52X4', 'H40.53X0', 'H40.53X1', 'H40.53X2', 'H40.53X3', 'H40.53X4', 'H40.60X0', 'H40.60X1', 'H40.60X2', 'H40.60X3', 'H40.60X4', 'H40.61X0', 'H40.61X1', 'H40.61X2', 'H40.61X3', 'H40.61X4', 'H40.62X0', 'H40.62X1', 'H40.62X2', 'H40.62X3', 'H40.62X4', 'H40.63X0', 'H40.63X1', 'H40.63X2', 'H40.63X3', 'H40.63X4', 'H40.811', 'H40.812', 'H40.813', 'H40.819', 'H40.821', 'H40.822', 'H40.823', 'H40.829', 'H40.831', 'H40.832', 'H40.833', 'H40.839', 'H40.89', 'H40.9', 'H42', 'H44.511', 'H44.512', 'H44.513', 'H44.519', 'H47.231', 'H47.232', 'H47.233', 'H47.239', 'Q15.0'

Heart failure: '398.91', '402.01', '402.11', '402.91', '404.01', '404.03', '404.11', '404.13', '404.91', '404.93', '428.0', '428.1', '428.20', '428.21', '428.22', '428.23', '428.30', '428.31', '428.32', '428.33', '428.40', '428.41', '428.42', '428.43', '428.9', 'I09.81', 'I11.0', 'I13.0', 'I13.2', 'I50.1', 'I50.20', 'I50.21', 'I50.22', 'I50.23', 'I50.30', 'I50.31', 'I50.32', 'I50.33', 'I50.40', 'I50.41', 'I50.42', 'I50.43', 'I50.810', 'I50.811', 'I50.812', 'I50.813', 'I50.814', 'I50.82', 'I50.83', 'I50.84', 'I50.89', 'I50.9'

Hyperlipidemia: '272.0','272.1', '272.2', '272.3', '272.4', 'E78.0', 'E78.00', 'E78.01', 'E78.1', 'E78.2', 'E78.3', 'E78.4', 'E78.5'

Hypertension: '362.11', '401.0', '401.1', '401.9', '402.00', '402.01', '402.10', '402.11', '402.90', '402.91', '403.00', '403.01', '403.10', '403.11', '403.90', '403.91', '404.00', '404.01', '404.02', '404.03', '404.10', '404.11', '404.12', '404.13', '404.90', '404.91', '404.92', '404.93', '405.01', '405.09', '405.11', '405.19', '405.91', '405.99', '437.2', 'H35.031', 'H35.032', 'H35.033', 'H35.039', 'I10', 'I11.0', 'I11.9', 'I12.0', 'I12.9', 'I13.0', 'I13.10', 'I13.11', 'I13.2', 'I15.0', 'I15.1', 'I15.2', 'I15.8', 'I15.9', 'I67.4', 'N26.2'

Ischemic heart disease: '410.00', '410.01', '410.02', '410.10', '410.11', '410.12', '410.20', '410.21', '410.22', '410.30', '410.31', '410.32', '410.40', '410.41', '410.42', '410.50', '410.51', '410.52', '410.60', '410.61', '410.62', '410.70', '410.71', '410.72', '410.80', '410.81', '410.82', '410.90', '410.91', '410.92', '411.0', '411.1', '411.81', '411.89', '412', '413.0', '413.1', '413.9', '414.00', '414.01', '414.02', '414.03', '414.04', '414.05', '414.06', '414.07', '414.12', '414.2', '414.3', '414.4', '414.8', '414.9', 'I20.0', 'I20.1', 'I20.8', 'I20.9', 'I21.01', 'I21.02', 'I21.09', 'I21.11', 'I21.19', 'I21.21', 'I21.29', 'I21.3', 'I21.4', 'I21.A1', 'I21.A9', 'I22.0', 'I22.1', 'I22.2', 'I22.8', 'I22.9', 'I23.0', 'I23.1', 'I23.2', 'I23.3', 'I23.4', 'I23.5', 'I23.6', 'I23.7', 'I23.8', 'I24.0', 'I24.1', 'I24.8', 'I24.9', 'I25.10', 'I25.110', 'I25.111', 'I25.118', 'I25.119', 'I25.2', 'I25.3', 'I25.41', 'I25.42', 'I25.5', 'I25.6', 'I25.700', 'I25.701', 'I25.708', 'I25.709', 'I25.710', 'I25.711', 'I25.718', 'I25.719', 'I25.720', 'I25.721', 'I25.728', 'I25.729', 'I25.730', 'I25.731', 'I25.738', 'I25.739', 'I25.750', 'I25.751', 'I25.758', 'I25.759', 'I25.760', 'I25.761', 'I25.768', 'I25.769', 'I25.790', 'I25.791', 'I25.798', 'I25.799', 'I25.810', 'I25.811', 'I25.812', 'I25.82', 'I25.83', 'I25.84', 'I25.89', 'I25.9'

Arthritis / rheumatoid arthritis: '714.0', '714.1', '714.2', '714.30', '714.31', '714.32', '714.33', '715.00', '715.04', '715.09', '715.10', '715.11', '715.12', '715.13', '715.14', '715.15', '715.16', '715.17', '715.18', '715.20', '715.21', '715.22', '715.23', '715.24', '715.25', '715.26', '715.27', '715.28', '715.30', '715.31', '715.32', '715.33', '715.34', '715.35', '715.36', '715.37', '715.38', '715.80', '715.89', '715.90', '715.91', '715.92', '715.93', '715.94', '715.95', '715.96', '715.97', '715.98', '720.0', '721.0', '721.1', '721.2', '721.3', '721.90', '721.91', 'M48.8X1', 'M48.8X2', 'M48.8X3', 'M48.8X4', 'M48.8X5', 'M48.8X6', 'M48.8X7', 'M48.8X8', 'M48.8X9' or diagnosis codes containing 'M05.', 'M06.', 'M08.', 'M15.', 'M16.', 'M17.', 'M18.', 'M19.', 'M45.', 'M47.'

Stroke: '430', '431', '433.01', '433.11', '433.21', '433.31', '433.81', '433.91', '435.0', '435.1', '435.3', '435.8', '435.9', '436', '997.02', 'G45.0', 'G45.1', 'G45.2', 'G45.8', 'G45.9', 'I61.0', 'I61.1', 'I61.2', 'I61.3', 'I61.4', 'I61.5', 'I61.6','I61.8', 'I61.9','I63.02', 'I63.00', 'I63.02', 'I63.03', 'I63.031', 'I63.032', 'I63.039', 'I63.09', 'I63.10', 'I63.111', 'I63.112', 'I63.119', 'I63.12', 'I63.131', 'I63.132', 'I63.139', 'I63.19', 'I63.6', 'I63.8', 'I63.9', 'I67.89', 'G97.3', 'I63.01' , 'I63.2', 'I63.3', 'I63.4', 'I63.5', 'I67.84', 'I97.81', 'I97.82' or diagnosis codes containing '434.', 'G46.', 'I60.', 'I66.'

(*If any of the qualifying claims also have an ICD-9 or and ICD-10 diagnosis code from the following list, then exclude:* 'S01.90XA', 'S02.0XXA', 'S02.0XXB', 'S02.10XA', 'S02.10XB', 'S02.101A', 'S02.101B', 'S02.102A', 'S02.102B', 'S02.109A', 'S02.109B', 'S02.11GA', 'S02.11GB', 'S02.11HA', 'S02.11HB', 'S02.110A', 'S02.111A', 'S02.112A', 'S02.113A', 'S02.110B', 'S02.111B', 'S02.112B', 'S02.113B', 'S02.118A', 'S02.118B', 'S02.119A', 'S02.119B', 'S02.19XA', 'S02.19XB', 'S02.2XXA', 'S02.2XXB', 'S02.3XXA', 'S02.30XA', 'S02.3XXB', 'S02.30XB', 'S02.31XA', 'S02.31XB', 'S02.32XA', 'S02.32XB', 'S02.40AA', 'S02.40AB', 'S02.40BA', 'S02.40BB', 'S02.40CA', 'S02.40CB', 'S02.40DA', 'S02.40DB', 'S02.40EA', 'S02.40EB', 'S02.40FA', 'S02.40FB', 'S02.400A', 'S02.400B', 'S02.401A', 'S02.401B', 'S02.402A', 'S02.402B', 'S02.411A', 'S02.411B', 'S02.412A', 'S02.412B', 'S02.413A', 'S02.413B', 'S02.42XA', 'S02.42XB', 'S02.600A', 'S02.600B', 'S02.601A', 'S02.601B', 'S02.602A', 'S02.602B', 'S02.609A', 'S02.609B', 'S02.61XA', 'S02.610A', 'S02.610B', 'S02.611A', 'S02.611B', 'S02.612A', 'S02.612B', 'S02.62XA', 'S02.620A', 'S02.62XB', 'S02.620B', 'S02.621A', 'S02.621B', 'S02.622A', 'S02.622B', 'S02.63XA', 'S02.630A', 'S02.63XB', 'S02.630B', 'S02.631A', 'S02.631B', 'S02.632A', 'S02.632B', 'S02.64XA', 'S02.640A', 'S02.64XB', 'S02.640B', 'S02.641A', 'S02.641B', 'S02.642A', 'S02.642B', 'S02.65XA', 'S02.650A', 'S02.65XB', 'S02.650B', 'S02.651A', 'S02.651B', 'S02.652A', 'S02.652B', 'S02.66XA', 'S02.66XB', 'S02.67XA', 'S02.670A', 'S02.670B', 'S02.671A', 'S02.671B', 'S02.672A', 'S02.672B', 'S02.69XA', 'S02.61XB', 'S02.62XA', 'S02.63XA', 'S02.64XA', 'S02.65XA', 'S02.66XA', 'S02.67XB', 'S02.69XB', 'S02.8XXA', 'S02.80XA', 'S02.8XXB', 'S02.80XB', 'S02.81XA', 'S02.81XB', 'S02.82XA', 'S02.82XB', 'S02.91XA', 'S02.91XB', 'S02.92XA', 'S02.92XB', 'S06.0X0A', 'S06.0X1A', 'S06.0X2A', 'S06.0X3A', 'S06.0X4A', 'S06.0X5A', 'S06.0X6A', 'S06.0X7A', 'S06.0X8A', 'S06.0X9A', 'S06.1X0A', 'S06.1X1A', 'S06.1X2A', 'S06.1X3A', 'S06.1X4A', 'S06.1X5A', 'S06.1X6A', 'S06.1X7A', 'S06.1X8A', 'S06.1X9A', 'S06.2X0A', 'S06.2X1A', 'S06.2X2A', 'S06.2X3A', 'S06.2X4A', 'S06.2X5A', 'S06.2X6A', 'S06.2X7A', 'S06.2X8A', 'S06.2X9A', 'S06.2X0B', 'S06.2X1B', 'S06.2X2B', 'S06.2X3B', 'S06.2X4B', 'S06.2X5B', 'S06.2X6B', 'S06.2X7B', 'S06.2X8B', 'S06.2X9B', 'S06.300A', 'S06.301A', 'S06.302A', 'S06.303A', 'S06.304A', 'S06.305A', 'S06.306A', 'S06.307A', 'S06.308A', 'S06.309A', 'S06.310A', 'S06.311A', 'S06.312A', 'S06.313A', 'S06.314A', 'S06.315A', 'S06.316A', 'S06.317A', 'S06.318A', 'S06.319A', 'S06.320A', 'S06.321A', 'S06.322A', 'S06.323A', 'S06.324A', 'S06.325A', 'S06.326A', 'S06.327A', 'S06.328A', 'S06.329A', 'S06.330A', 'S06.331A', 'S06.332A', 'S06.333A', 'S06.334A', 'S06.335A', 'S06.336A', 'S06.337A', 'S06.338A', 'S06.339A', 'S06.340A', 'S06.341A', 'S06.342A', 'S06.343A', 'S06.344A', 'S06.345A', 'S06.346A', 'S06.347A', 'S06.348A', 'S06.349A', 'S06.350A', 'S06.351A', 'S06.352A', 'S06.353A', 'S06.354A', 'S06.355A', 'S06.356A', 'S06.357A', 'S06.358A', 'S06.359A', 'S06.360A', 'S06.361A', 'S06.362A', 'S06.363A', 'S06.364A', 'S06.365A', 'S06.366A', 'S06.367A', 'S06.368A', 'S06.369A', 'S06.370A', 'S06.371A', 'S06.372A', 'S06.373A', 'S06.374A', 'S06.375A', 'S06.376A', 'S06.377A', 'S06.378A', 'S06.379A', 'S06.380A', 'S06.381A', 'S06.382A', 'S06.383A', 'S06.384A', 'S06.385A', 'S06.386A', 'S06.387A', 'S06.388A', 'S06.389A', 'S06.4X0A', 'S06.4X1A', 'S06.4X2A', 'S06.4X3A', 'S06.4X4A', 'S06.4X5A', 'S06.4X6A', 'S06.4X7A', 'S06.4X8A', 'S06.4X9A', 'S06.5X0A', 'S06.5X1A', 'S06.5X2A', 'S06.5X3A', 'S06.5X4A', 'S06.5X5A', 'S06.5X6A', 'S06.5X7A', 'S06.5X8A', 'S06.5X9A', 'S06.6X0A', 'S06.6X1A', 'S06.6X2A', 'S06.6X3A', 'S06.6X4A', 'S06.6X5A', 'S06.6X6A', 'S06.6X7A', 'S06.6X8A', 'S06.6X9A', 'S06.810A', 'S06.811A', 'S06.812A', 'S06.813A', 'S06.814A', 'S06.815A', 'S06.816A', 'S06.817A', 'S06.818A', 'S06.819A', 'S06.820A', 'S06.821A', 'S06.822A', 'S06.823A', 'S06.824A', 'S06.825A', 'S06.826A', 'S06.827A', 'S06.828A', 'S06.829A', 'S06.890A', 'S06.891A', 'S06.892A', 'S06.893A', 'S06.894A', 'S06.895A', 'S06.896A', 'S06.897A', 'S06.898A', 'S06.899A', 'S06.9X0A', 'S06.9X1A', 'S06.9X2A', 'S06.9X3A', 'S06.9X4A', 'S06.9X5A', 'S06.9X6A', 'S06.9X7A', 'S06.9X8A', 'S06.9X9A' or diagnosis codes containing '804.9', '850', '854.1', 'V57.', 'Z51.89')

Anxiety: '293.84', '300.00', '300.01', '300.02', '300.09', '300.10', '300.20', '300.21', '300.22', '300.23', '300.29', '300.3', '300.5', '300.89', '300.9', '308.0', '308.1', '308.2', '308.3', '308.4', '308.9', '309.81', '313.0', '313.1', '313.21', '313.22', '313.3', '313.82', '313.83', 'F06.4', 'F40.00', 'F40.01', 'F40.02', 'F40.10', 'F40.11', 'F40.210', 'F40.218', 'F40.220', 'F40.228', 'F40.230', 'F40.231', 'F40.232', 'F40.233', 'F40.240', 'F40.241', 'F40.242', 'F40.243', 'F40.248', 'F40.290', 'F40.291', 'F40.298', 'F40.8', 'F40.9', 'F41.0', 'F41.1', 'F41.3', 'F41.8', 'F41.9', 'F42', 'F42.2', 'F42.3', 'F42.4', 'F42.8', 'F42.9', 'F43.0', 'F43.10', 'F43.11', 'F43.12', 'F44.9', 'F45.8', 'F48.8', 'F48.9', 'F93.8', 'F99', 'R45.2', 'R45.5', 'R45.6', 'R45.7'

Depressive disorders: '296.20', '296.21', '296.22', '296.23', '296.24', '296.25', '296.26', '296.30', '296.31', '296.32', '296.33', '296.34', '296.35', '296.36', '300.4', '311', 'V79.0', 'F32.0', 'F32.1', 'F32.2', 'F32.3', 'F32.4', 'F32.5', 'F32.89', 'F32.9', 'F33.0', 'F33.1', 'F33.2', 'F33.3', 'F33.8', 'F33.40', 'F33.41', 'F33.42', 'F33.9', 'F34.1'

Fibromyalgia / Chronic pain / Fatigue: '338.2', '338.21', '338.22', '338.23', '338.29', '338.3', '338.4', '780.7', '780.71', '729.1', '729.2', 'G89.21', 'G89.22', 'G89.28', 'G89.29', 'G89.3', 'G89.4', 'M54.10', 'M54.11', 'M54.12', 'M54.13', 'M54.14', 'M54.15', 'M54.16', 'M54.17', 'M54.18', 'M60.80', 'M60.811', 'M60.812', 'M60.819', 'M60.821', 'M60.822', 'M60.829', 'M60.831', 'M60.832', 'M60.839', 'M60.841', 'M60.842', 'M60.849', 'M60.851', 'M60.852', 'M60.859', 'M60.861', 'M60.862', 'M60.869', 'M60.871', 'M60.872', 'M60.879', 'M60.88', 'M60.89', 'M60.9', 'M79.1', 'M79.2', 'M79.7', 'R53.82'

Leukemia / lymphoma: 'C88.4', 'C96.4', 'C96.9', 'C96.Z', 'D45', 'Z85.231', 'Z85.6', 'Z85.71', 'Z85.79', '203.1', 'V10.6', 'V10.7', 'C90.1' or diagnosis codes containing '200.', '201.', '202.', '204.', '205.', '206.', '207.', '208.', 'C81.', 'C82.', 'C83.', 'C84.', 'C85.', 'C86.', 'C91.', 'C92.', 'C93.', 'C94.', 'C95.'

Liver disease: '571.8', '571.9', 'K75.89', 'K74.0', 'K74.1', 'K74.2', 'K76.0', 'K75.81'

Gallbladder disease: '576.1','K80.30', 'K80.31','K80.32', 'K80.33', 'K80.34', 'K80.35', 'K80.36', 'K80.37', 'K83.0'

Migraine: diagnosis codes containing '339.', '346.', 'G43.', 'G44.'

Mobility impairments: '438.2', '438.3', '438.4', '438.5', 'I69.03', 'I69.04', 'I69.05', 'I69.06', 'I69.13', 'I69.14', 'I69.15', 'I69.16', 'I69.23', 'I69.24', 'I69.25', 'I69.26', 'I69.33', 'I69.34', 'I69.35', 'I69.36', 'I69.83', 'I69.84', 'I69.85', 'I69.86', 'I69.93', 'I69.94', 'I69.95', 'I69.96', '334.1', 'G04.1', 'G11.4' or diagnosis codes containing '344', '342.', '344.', 'G81.', 'G82.', 'G83.'

Peripheral artery disease: '440.0', '440.1', '440.2', '440.20', '440.21', '440.22', '440.23', '440.29', '440.4', '443.8', '443.81', '443.82', '443.89', '443.9', 'E08.51', 'E08.52', 'E09.51', 'E09.52', 'E10.51', 'E10.52', 'E11.51', 'E11.52', 'E13.51', 'E13.52', 'I70.0', 'I70.1', 'I70.201', 'I70.202', 'I70.203', 'I70.208', 'I70.209', 'I70.211', 'I70.212', 'I70.213', 'I70.218', 'I70.219', 'I70.221', 'I70.222', 'I70.223', 'I70.228', 'I70.229', 'I70.231', 'I70.232', 'I70.233', 'I70.234', 'I70.235', 'I70.238', 'I70.239', 'I70.241', 'I70.242', 'I70.243', 'I70.244', 'I70.245', 'I70.248', 'I70.249', 'I70.25', 'I70.291', 'I70.292', 'I70.293', 'I70.298', 'I70.299', 'I70.92', 'I73.81', 'I73.89', 'I73.9', 'I79.1', 'I79.8'

Pressure and chronic ulcers: 'I70.25', 'I70.75', 'I70.23', 'I70.23', 'I70.24', 'I70.33', 'I70.34', 'I70.43', 'I70.44', 'I70.53', 'I70.54', 'I70.63', 'I70.64', 'I70.73', 'I70.74', 'I70.64', 'L98.4' or diagnosis codes containing '707.', 'L89.', 'L97.'

Obstructive sleep apnea: '327.23', '780.57', 'G47.33', 'G47.30'

Chronic lower back pain: '720', '720.1', '720.2', '721.3', '721.42', '722.10', '722.11', '722.32', '722.51', '722.52', '722.73', '722.83', '722.93', '724', '724.02', '724.2', '724.3', '724.4', '724.5', '724.6', '724.70', '724.71', '724.79', '738.4', '739.3', '739.4', '756.11', '756.12', '805.4', '805.6', '846', '846.1', '846.2', '846.3', '846.8', '846.9', '847.2', '847.3', '847.4', 'M46.00', 'M46.1', 'M47.817', 'M47.16', 'M51.26', 'M51.27', 'M51.24', 'M51.25', 'M51.46', 'M51.47', 'M51.34', 'M51.35', 'M51.36', 'M51.37', 'M51.06', 'M96.1', 'M46.47', 'M51.86', 'M51.87', 'M48.061', 'M54.5', 'M54.30', 'M54.14', 'M54.15', 'M54.16', 'M54.17', 'M54.89', 'M54.9', 'M43.27', 'M43.28', 'M53.2X7', 'M53.3', 'M53.2X8', 'M43.00', 'M43.10', 'M99.03', 'M99.04', 'Q76.2', 'S32.009A', 'S32.10XA', 'S32.2XXA', 'S33.8XXA', 'S33.6XXA', 'S33.9XXA', 'S33.8XXA', 'S33.8XXA', 'S33.5XXA', 'S33.8XXA', 'S33.8XXA'

Type 2 diabetes: '250.00','250.02', '250.10','250.12', '250.20','250.22', '250.30','250.32', '250.40','250.42', '250.50','250.52', '250.60', '250.62','250.70','250.72','250.80','250.82','250.90','250.92', 'E11' or diagnosis codes containing 'E11.'
